# Nonsense Mutation in Coiled-Coil Domain Containing 151 Gene (*CCDC151*) Causes Primary Ciliary Dyskinesia

**DOI:** 10.1002/humu.22698

**Published:** 2014-09-16

**Authors:** Muslim M Alsaadi, A Mesut Erzurumluoglu, Santiago Rodriguez, Philip A I Guthrie, Tom R Gaunt, Hager Z Omar, Mohammad Mubarak, Khalid K Alharbi, Ammar C Al-Rikabi, Ian N M Day

**Affiliations:** 1 College of Medicine, King Saud UniversityRiyadh, 11533, Kingdom of Saudi Arabia; 2 Bristol Genetic Epidemiology Laboratories (BGEL), University of Bristol, Bristol BS8 2BNUnited Kingdom; 3 MRC Integrative Epidemiology Unit (IEU), School of Social and Community Medicine, University of BristolBristol, BS8 2BN, United Kingdom; 4 Histopathology Unit, Department of Pathology & Laboratory Medicine, College of Medicine and King Khalid University Hospital, King Saud UniversityRiyadh, 11461, Kingdom of Saudi Arabia; 5 Clinical Laboratory Sciences Department, College of Applied Medical Sciences, King Saud UniversityRiyadh, 11433, Kingdom of Saudi Arabia

**Keywords:** primary ciliary dyskinesia, *CCDC151*, respiratory cilia, ciliopathy

## Abstract

Primary ciliary dyskinesia (PCD) is an autosomal-recessive disorder characterized by impaired ciliary function that leads to subsequent clinical phenotypes such as chronic sinopulmonary disease. PCD is also a genetically heterogeneous disorder with many single gene mutations leading to similar clinical phenotypes. Here, we present a novel PCD causal gene, coiled-coil domain containing 151 (*CCDC151*), which has been shown to be essential in motile cilia of many animals and other vertebrates but its effects in humans was not observed until currently. We observed a novel nonsense mutation in a homozygous state in the *CCDC151* gene (NM_145045.4:c.925G>T:p.[E309*]) in a clinically diagnosed PCD patient from a consanguineous family of Arabic ancestry. The variant was absent in 238 randomly selected individuals indicating that the variant is rare and likely not to be a founder mutation. Our finding also shows that given prior knowledge from model organisms, even a single whole-exome sequence can be sufficient to discover a novel causal gene.

Primary ciliary dyskinesia (PCD; MIM #244400) is a genetically heterogeneous ciliopathy inherited in an autosomal-recessive fashion [Bush et al., 2007], although there have been a few instances where PCD has been reported to follow an autosomal-dominant inheritance pattern [Narayan et al., 1994; Alvarez Gonzalez et al., 2006]. PCD is a rare (∼one in 20,000) disorder associated with abnormal (respiratory) ciliary structure and/or function, abnormal sperm motility (and therefore infertility or later subfertility), and/or situs abnormalities [Zariwala et al., 1993]. The respiratory ciliary pathology causes chronic airway infections due to retention of mucus (and bacteria) in the respiratory tract [Zariwala et al., 1993].

Understanding the genetic aetiology of PCD has been challenging. In 2007, only five genes (*DNAI1*, *DNAH5*, *DNAH11*, *RPGR*, and *OFD1*) had been identified and these accounted for just 40% of the cases [Zariwala et al., 1993]. However, the last 6 years has yielded a plethora of human PCD causal genes (adding over 20 genes and two linkage regions, CILD4 and CILD8), mostly due to whole-exome sequencing (WES) technologies becoming widespread, which enabled hypothesis-free and cost-efficient searching for novel genes [Zariwala et al., 1993; Moore et al., 2013]. However, these genes still only account for approximately 70% of PCD cases (although difficult to state a confident figure at present) [Zariwala et al., 1993]. It can be anticipated that there will be considerable further locus heterogeneity in the remaining 30% or more, bearing in mind the number of proteins constituting the structure and function of motile cilia. In this study, we analyzed the exome of an individual from a consanguineous family who was clinically diagnosed with PCD. Especially studying the autozygous regions, where causal variants are mainly found, we identified human *CCDC151* deficiency as a further cause of PCD.

A male patient from a consanguineous family of Arabic descent with clinical features consistent with PCD such as bronchiectasis, bronchial asthma, recurrent chest infections, low nasal nitric oxide levels (measurement: 5), and a long history of wet cough with light-green colored sputum (also allergic rhinitis and pectus excavatum) and his unaffected brother were studied (see Supp. Materials and Methods for ethical consent). The parents’ blood samples were also collected for further analysis. Details on all methods used in this study can be found in the Supp. Materials and Methods. Summary statistics from WES of proband can be found in Supp. Results.

Initially, all LRoH regions identified by Plink [Purcell et al., 2007] and our custom Python script were reviewed in IGV [Thorvaldsdottir et al., 2013] to check whether any known PCD gene (or reported linkage region) resides within the region. None of these regions spanned a known human PCD gene or a reported linkage region. Several filters (e.g., minor allele frequency [MAF], consequence of variant) were applied systematically on all mutations to single out any potentially causal ones. All “predicted high impact” (hereafter Φ) mutations (i.e., rare stop gains/losses, start losses, splice-site acceptor/donor variants, missense mutations, indels—both nonframeshifting and frameshifting) that were in a homozygous state, were analyzed separately in the two candidate lists. This yielded only nine homozygous Φ variants in list 1 (i.e., known genes, see Supp. Materials and Methods) and a total of 349 Φ variants in list 2 (i.e., all suspected ciliome genes; see Supp. Materials and Methods). However, all the mutations in list 1 were common (i.e., with MAF = >1%) and just two of the mutations in list 2 passed this MAF criteria, which were a stop gained (c.925G>T:p.[E309*], see Supp. Fig. S1) in the *CCDC151* gene and the other was a frameshifting insertion in the *ZNF595* gene (p.N201fs). The stop gain was absent in dbSNP, Exome Variant Server (EVS), our internal database (which includes his unaffected sibling), and 1000 Genomes Project databases, whereas the insertion was present (in a homozygous state) in many of our previously whole-exome sequenced controls (of Arabic ancestry) and also had a total MAF of 8.6% in EVS. The filtering process is depicted in Figure1. The stop gain was located within a long LRoH region (∼17 Mb) on chromosome 19. The mutation falls near the center of the protein (which is 595 amino acids long) and resides in a highly conserved region represented by a (36-way Eutherian mammals) GERP score of 245.1 (also see Supp. Table S1 for local alignments) [Davydov et al., 2010]. A final analysis was carried out on all the mutations outside of the two candidate lists and no other mutations were identified that passed all the filtering criteria used in Figure1. The additional CNV analysis yielded no apparent gains or losses in the genes contained in the two candidate gene lists. The variant has been submitted to LOVD3 database (http://databases.lovd.nl/shared/variants/0000040146#04563).

**Figure 1:**
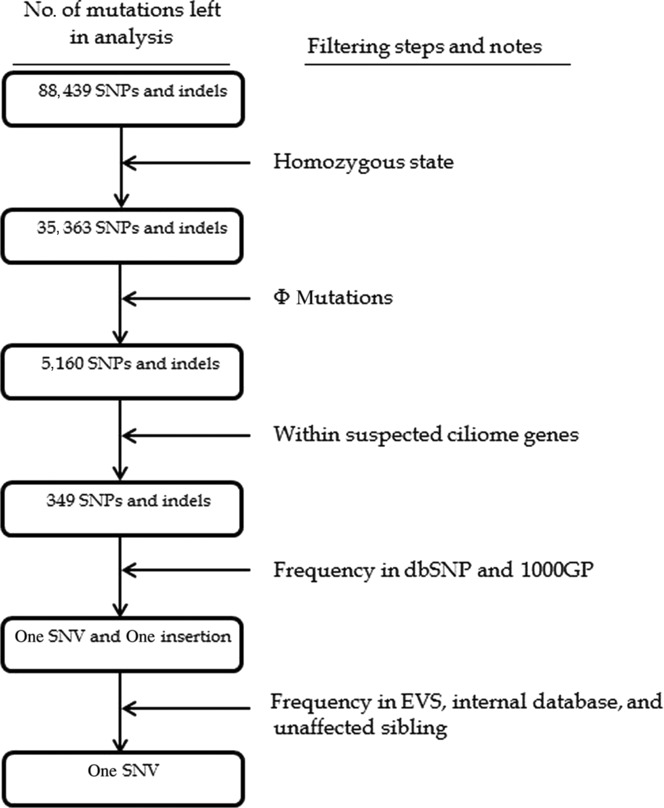
Filtering steps applied to all mutations in the exome. After all the filtering steps in the above figure were applied, the total was reduced to a single one in *CCDC151* (GenBank reference sequence: NM_145045.4). Φ mutations: rare stop gains/losses, start losses, splice-site acceptor/donor variants, missense mutations, and exonic indels (see Supp. Materials and Methods for details).

PCR was used to amplify a region 221 bp long (harboring the stop gain) in the proband, the unaffected brother, and the parents, which was then subsequently sequenced (using Sanger sequencing method) and digested with AvrII enzyme to confirm the variant status (Supp. Tables S2 and 3; Supp. Fig. S2). As expected, the parents are heterozygous and the proband is homozygous in accordance with autosomal-recessive mode of inheritance of PCD. The unaffected brother is also heterozygous.

To deduce how common the c.925G>T:p.(E309*) variant in *CCDC151* (GenBank reference sequence: NM_145045.4) is in the local population, a buccal swab sample from 238 randomly selected individuals of Saudi Arabian ancestry (male and female, living in Riyadh) were collected (see Supp. Materials and Methods for details). The PCR amplicons produced (using primers in Supp. Table S2) were digested using the AvrII enzyme and viewed using 96-well MADGE to check for presence of the p.(E309*) variant. None of the 238 wells showed any digestion (Supp. Figure S3A–C).

*CCDC151* gene was first identified as a potential human ciliome gene by Ostrowski et al. (2002). The expressed sequence tag of *CCDC151* was among the 110 proteins (with Accession no: BAB01602) identified by one-dimensional polyacrylamide gel electrophoresis analysis of human ciliary axonemes (an additional 104 additional proteins were identified using different methods). More recently, Jerber et al. (2014) carried out functional analyses of *CCDC151* in *Drosophila*, mice, and zebrafish. They showed that *CCDC151* was associated with motile intraflagellar transport (IFT)-dependent cilia in *Drosophila*. In the same analysis, they reported that *Ccdc151* was expressed in tissues with motile cilia in zebrafish, and morpholino-induced depletion of the gene product lead to, similar to human PCD phenotypes, left–right asymmetry defects [Jerber et al., 2014]. They demonstrated that *Ccdc151* is strongly expressed in (and was restricted to) motile-ciliated tissues, where it is required for dynein arm assembly and for the transport of the docking complex *Ccdc114* (homolog of a known human PCD causal gene) [Jerber et al., 2014]. It was also required for proper motile function of cilia in the Kupffer's vesicle (a ciliated organ in the zebrafish embryo that initiates left–right development of the brain, heart, and gut [Essner et al., 2005]) and in the pronephros (an excretory organ in vertebrates) by controlling dynein arm assembly, showing that *Ccdc151* is important in the control of IFT-dependent dynein arm assembly in many animals [Jerber et al., 2014]. Furthermore, knockdown of *Ccdc151* in IMCD3 mouse cells resulted in a deregulated ciliary length [Jerber et al., 2014]. A similar analysis was carried out by Dean and Mitchell (2013) in *Chlamydomonas* ODA10 gene that is the homolog of the mouse Ccdc151 gene (very similar to the human *CCDC151* gene, see Supp. Table S1) and was found to play an important role in the outer dynein arm assembly.

Consistent with the abovementioned zebrafish study [Jerber et al., 2014], the cilia structure of the *CCDC151* mutant cilia from the PCD patient is strikingly similar to the *ccdc151* morphant cilia (see Fig. 5F of [Jerber et al., 2014]) where the axonemes do not assemble a full complement of ODA and IDA (Fig.2). These observations support the essential role *CCDC151* plays for targeting of dynein arms and the dynein arm–docking complex to the axoneme [Jerber et al., 2014].

**Figure 2:**
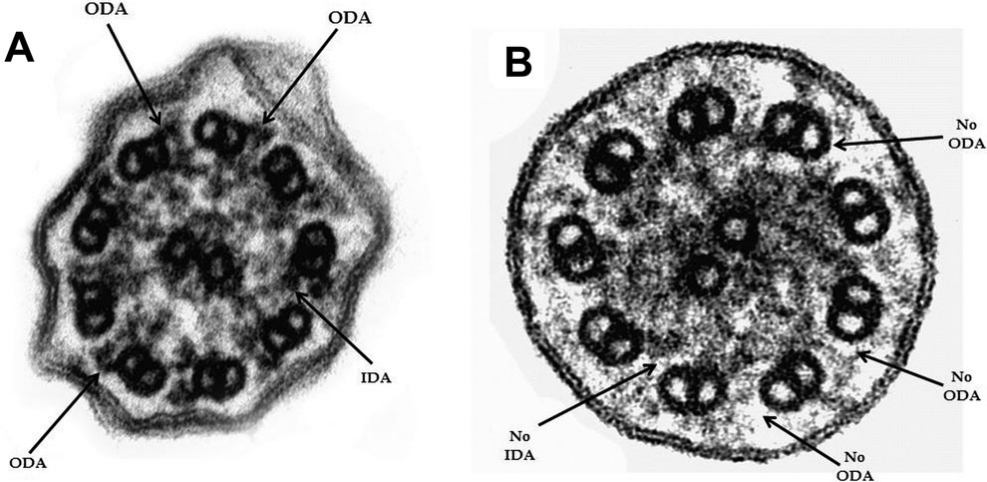
Cross-sections of respiratory cilia in (A) control and (B) *CCDC151* mutated proband (*n* = 247). The ultrastructural EM images of the cilia confirms the absence of IDA and ODA in the *CCDC151* mutant cilia (74% of 247 cilia scored)—similar to the *ccdc151* morphant in Jerber et al. (2014). For notes on cilia beating, see Supp. Material and Methods. EM magnification: 250,000×.

An additional statistical analysis in a “frequentist” manner was carried out in order to quantify the probability of this mutation being a null finding. For this, the number of very rare (or non-dbSNP) Φ mutations in autosomes were quantified in three healthy individuals (two of Arabic and one of European ancestry); and this figure was found to be approximately 60 on average. However, a very large proportion of these were in a heterozygous state and in olfactory, taste- and hearing-related genes. The amount of Φ mutations that fell in the entire ciliome database (i.e. list 1 and 2) was on average one. The ciliome database contains within it 2,004 genes related to all types of cilia (motile and nonmotile, and in different tissues), but a crude estimate on the number of “respiratory cilia”-related genes (not including the known PCD genes) was estimated to be approximately 100 (i.e., second-tier candidate human PCD genes). As aforementioned, known PCD genes explain around 70% of PCD, which leaves the other 30% to these 100 genes. Thus, the probability of one of these Φ mutations falling in one of the second-tier candidate PCD genes in an autozygous region (inbreeding coefficient of offspring of first cousins, *F* = 1/16) was calculated to be approximately 0.0009 (i.e., Pr = 1/16 × 100/2004 × 1 × 0.30). Additional evidence from the absence of mutation in EVS and internal database (which includes the unaffected sibling and individuals of Arabic ancestry), GERP scores [Davydov et al., 2010], consequence of variant (i.e., a stop gain near the center of the protein where it affects all transcripts of the gene), and functional analyses of *CCDC151* carried out by Jerber et al. (2014) and Dean and Mitchell (2013) are additional and significant points to reject the null hypothesis, but since it is hard to quantify these statistically (except to note that the animal functional studies are very compelling), we cannot incorporate these points into the likelihood calculation. Thus, the c.925G>T: p.(E309*) variant in *CCDC151* is very likely to be the causal variant.

Although many PCD causal genes have been identified in humans, a comprehensive PCD gene interactome has not yet been established. Moreover, the molecular mechanisms of how mutations in certain PCD causal genes also cause laterality defects (e.g., situs inversus), certain combinations of ultrastructural defects and/or sterility (or subfertility) are far from being completely understood [Vincensini et al., 2011]. However, identifying all PCD causal genes will contribute toward understanding the mechanisms (e.g., interactome of PCD causal pathway) behind these various clinical phenotypes and speed up the initiation of improved intervention trials to treat this complex disorder.

To conclude, although animal models have strongly linked *CCDC151* to cilia [Jerber et al., 2014], complete human inactivations of *CCDC151* were not observed (until now) to associate these findings to the human model. Here, we report a homozygous nonsense mutation (c.925G>T, p.[E309*]) in *CCDC151* in a patient who has been clinically diagnosed with PCD. The variant was screened in 238 unrelated individuals, and it was found to be absent in all of them indicating that the mutation is not a founder mutation and may have occurred relatively recently and/or is tribal specific. Nevertheless, *CCDC151* adds to the already identified 25 genes (with six of them being coiled-coil domain containing genes: *CCDC39*, *CCDC40*, *CCDC65*, *CCDC103*, *CCDC114*, and *CCDC164*) in which mutations are known to cause PCD and indicates the important role *CCDC151* plays in human respiratory ciliary function. Our findings also show that given prior knowledge from an animal model, even a single WES (with high read depth) can be adequate when pinpointing a novel causal gene.

## Supporting Information

Disclaimer: Supplementary materials have been peer-reviewed but not copyedited.
