# Supplementary material for: Nonsense Mutation in Coiled-Coil Domain Containing 151 Gene (CCDC151) Causes Primary Ciliary Dyskinesia
Source: Hum Mutat. 2014 Sep 16;35(12):1446–8. doi: 10.1002/humu.22698 (PMC4489323; doi:10.1002/humu.22698)
Supplement: Supplementary file 1 — Supporting Information [file humu0035-1446-sd1.pdf]

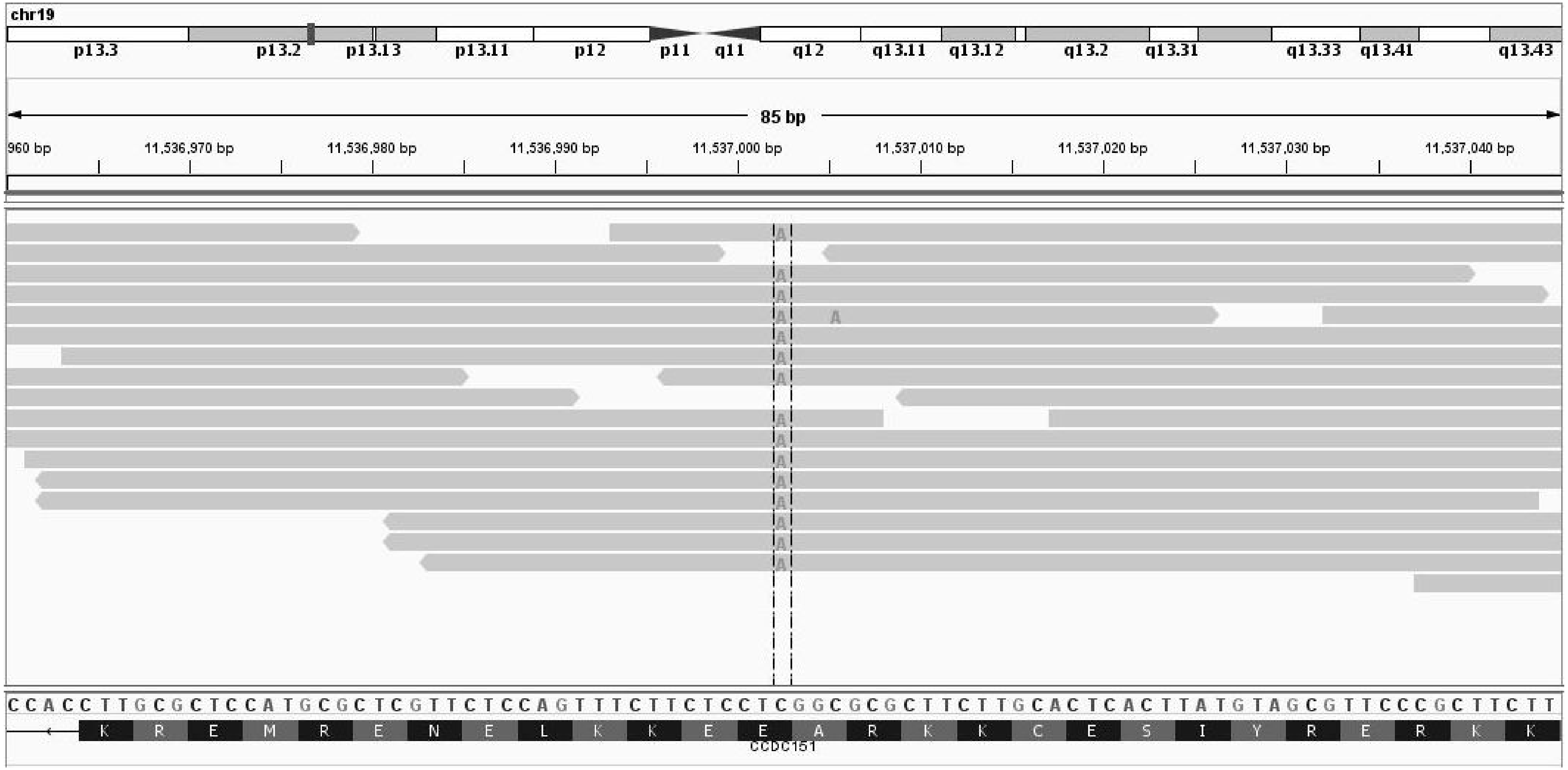

Supp. Figure S1. Reads mapped to the reference human genome hg19 at the site of the c.924C>A (p.E309*) mutation. All fifteen reads are high quality and there are no wild type alleles. The image was created using IGV. NB: The *CCDC151* gene (GenBank reference sequence: NM_145045.4) is on the reverse strand, therefore the sequence (shown here) must be reversed (e.g. the mutation is G>T causing GAG to become TAG, a premature stop codon). This variant status (i.e. homo/heterozygosity) was confirmed using Sanger sequencing and AvrII digestion in the proband, parents and the unaffected brother (Supp. Figure S2**).** Nucleotide numbering system uses +1 as the A of the ATG translation initiation codon in the reference sequence, with the initiation codon (Met) as codon 1.

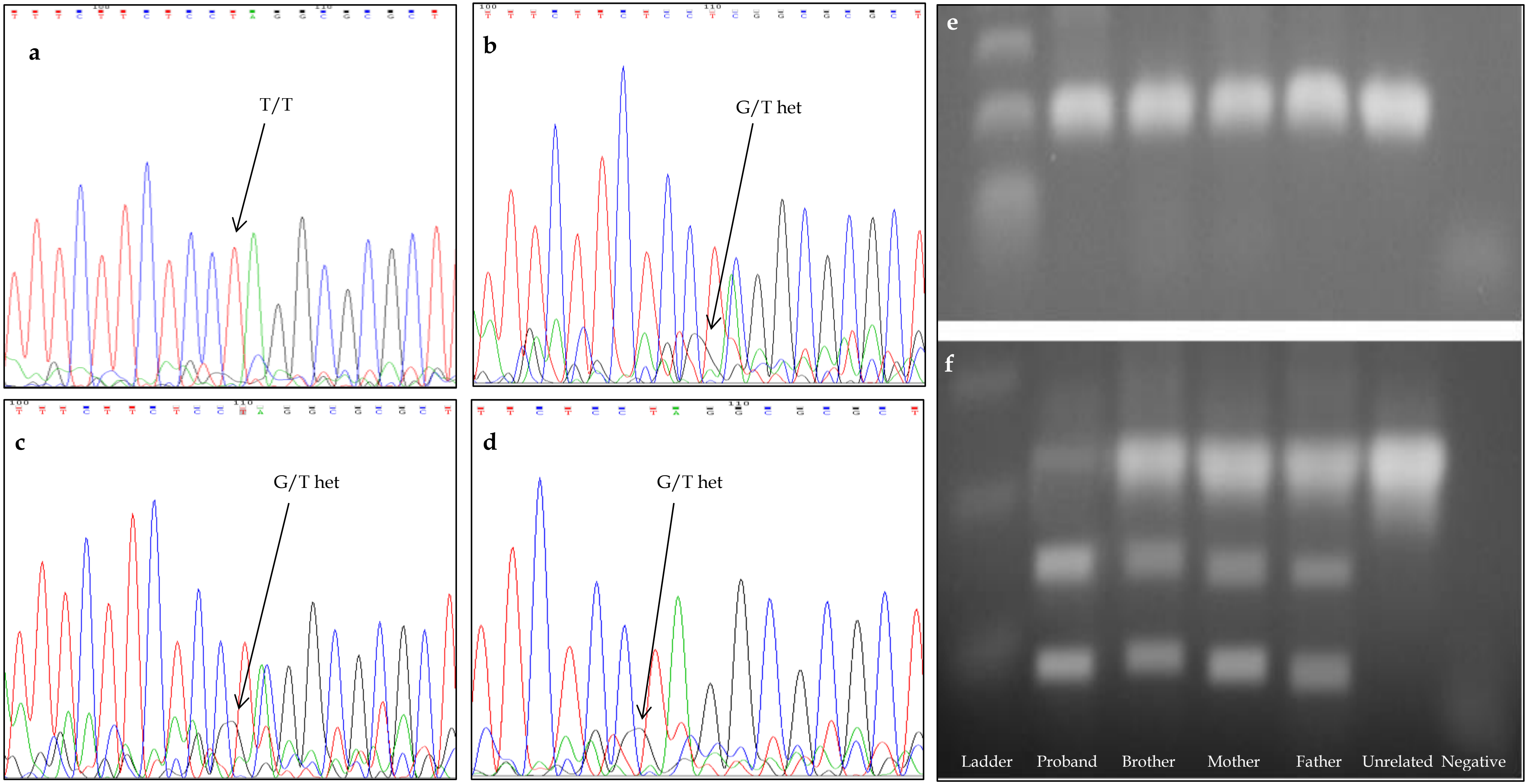

Supp. Figure S2 Confirmation of variant status in proband and other family members using (a-d) *Sanger sequencing and (e-f) AvrII digestion. (a) Proband (b) Unaffected brother (c) Mother (d) Father (e) PCR amplicons before restriction enzyme digestion (f) After digestion. Ladder: 300bp (top), 200bp and 100bp (bottom). DNA Chromatogram images were created using Chromas Lite (v2.1.1). *Peak height imbalances could have been caused by low template DNA, degraded DNA and/or preferential amplification.

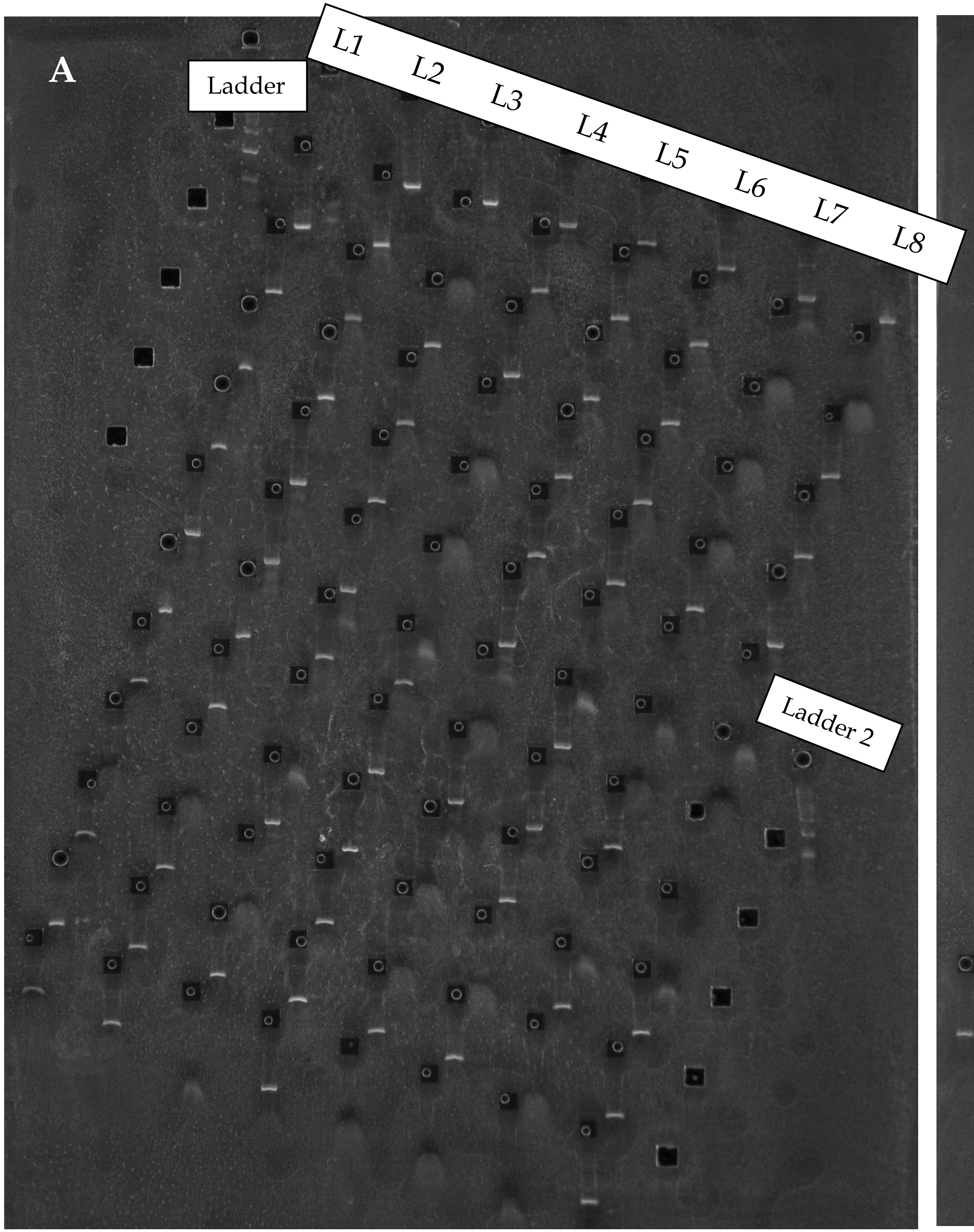
A
Ladder
L1
L2
L3
L4
L5
L6
L7
L8
Ladder 2

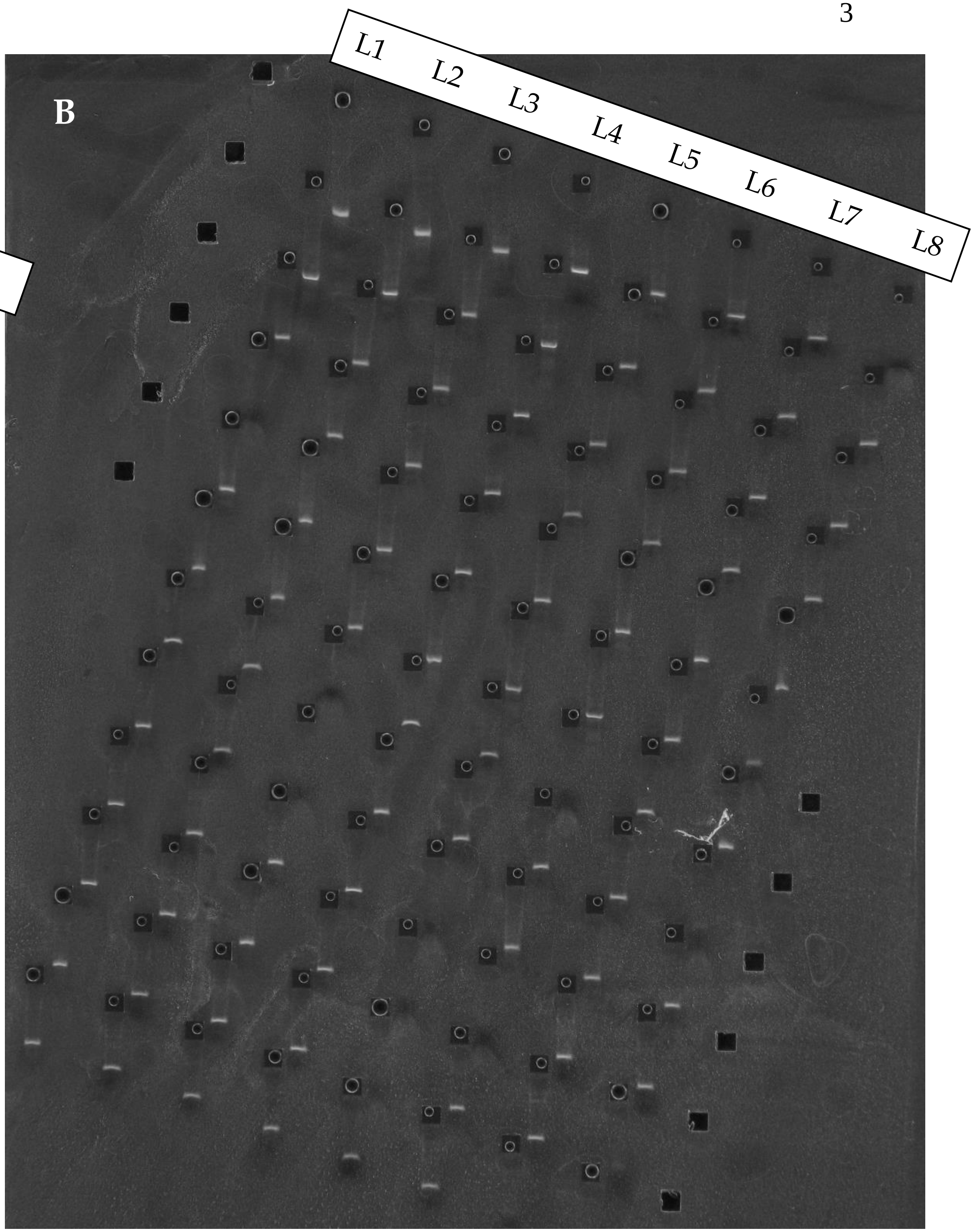
B
L1
L2
L3
L4
L5
L6
L7
L8

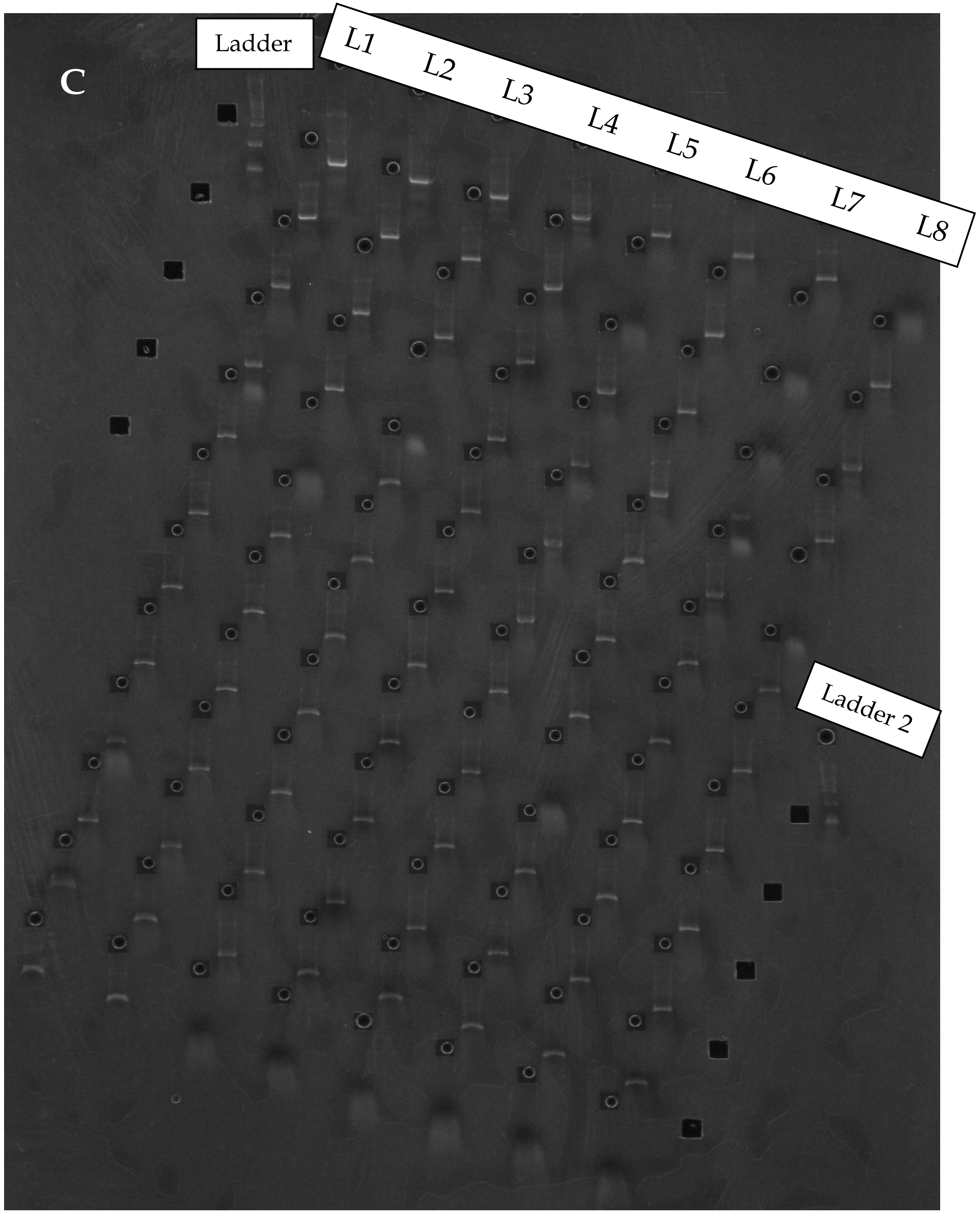

Supp. Figure S3A-C. Screening the Saudi population for the p.E309X variant. 96-well MADGE images reveal that none of the 238 individuals have the causal allele. Ladders last three bands are 100bp (bottom), 200bp and 300bp (top).

Supp. Table S1. Local sequence alignment containing the mutated residue from multiple alignment of the *CCDC151* gene in different organisms (relevant species shown)

Start	Amino acid sequence	End	Entry	Entry Name	Organism
298	ERYISECKKRAEEKKLENERMERK	321	A5D8V7	CC151_HUMAN	Homo sapiens (Human)
294	EHYITDCKKRAEEKKLQTERMERK	317	G3X951	G3X951_MOUSE	Mus musculus (Mouse)
298	ERYISECKKRAEEKKLENERMERK	321	F6XD06	F6XD06_MACMU	Macaca mulatta (Rhesus macaque)
277	ERYISECKKRAEEKKLENERMERK	300	H2QFD9	H2QFD9_PANTR	Pan troglodytes (Chimpanzee)
297	ECYISECKKRAEERKLENQRMERK	320	F7IJB7	F7IJB7_CALJA	Callithrix jacchus (White-tufted-ear marmoset)
298	ERYVTECKKRAEEKKLENERMERK	321	H0XEE5	H0XEE5_OTOGA	Otolemur garnettii (Small-eared galago)
300	ERFISDCKKRAEEKKLQNERMERK	323	I3NHF3	I3NHF3_SPETR	Spermophilus tridecemlineatus (13-lined ground squirrel)
298	ERYLTECKKRAEEKKLQNERMERK	321	A7MBH5	CC151_BOVIN	Bos taurus (Bovine)
298	ERYITECKKRAEDRKLQNERMERK	321	E2RKK3	E2RKK3_CANFA	Canis familiaris (Dog) (Canis lupus familiaris)
298	ERYITECKKRAEERKLQNERMERK	321	G1LVJ7	G1LVJ7_AILME	Ailuropoda melanoleuca (Giant panda)
298	ERYITECKKRAEDRKLQNERMERK	321	M3Y1B7	M3Y1B7_MUSPF	Mustela putorius furo (European domestic ferret)
247	ETALTELKAQAEEKKAHAERVERR	270	Q2PEE6	Q2PEE6_CIOIN	Ciona intestinalis (Transparent sea squirt)
265	ERQALDFRKQVEARKLELERIGRK	288	B4PFS1	B4PFS1_DROYA	Drosophila yakuba (Fruit fly)
204	EFYITDCKKRAEEKKLQTERMERK	227	G3H698	G3H698_CRIGR	Cricetulus griseus (Chinese hamster)

The highlighted Glutamic acid (E) residue is found to be highly conserved across many species where the gene is predicted to be a homologue of the human *CCDC151* gene. The alignment was carried out using the Uniprot website's Blast and Align functions (http://www.uniprot.org)

Supp. Table S2. Primers used to amplify a 221bp long region containing the p.E309X mutation in the *CCDC151* gene

**Primer**	**Base**
Forward	5'-AAATGGGAGAAGGCCTAGGATG-3'
Reverse	5'-GAACCAGCTGCAGTACCTAGAG-3'

Supp. Table S3. The AvrII enzyme will digest the PCR amplicons produced using the primers in Supp. Table S1 where it comes across the sequence CCTAGG (cutting between the two cytosine bases)

Enzyme	Cut site	Unaffected	Affected	Size of PCR fragment for unaffected after digestion	Sizes of PCR fragments for affected after digestion
AvrII	5′-C|CTAGG-3′	5′-GCCGAGGAG-3′	5′-GCCTAGGAG-3′	221bp	84bp and 137bp

Since the unaffected individuals will not have the thymine base required for digestion, the PCR amplicons will stay unaltered (i.e. 221bp long).

Supp. Materials and Methods

Ethical approval and consents

Ethical approval was obtained from the King Saud University/King Khalid Hospital, Riyadh ethical committee (approval number: E-11-448). Family and individual consent was verbal, with the recognition that positive findings would be diagnostically reconfirmed in conjunction with clinical counselling and feedback.

For the mutation screening samples (238 individuals), inclusion was voluntary, and informed written consent for anonymised genetic studies was taken in keeping with King Saud University College of Applied Medical Sciences guidelines.

Participants and Genetic Data Analysis

DNA was extracted from peripheral blood using the QIAamp DNA Mini kit provided by QIAGEN (Catalogue No: 51304); and the protocol for DNA Purification from Blood or Body Fluids was followed in the QIAamp DNA Mini and Blood Mini handbook. The exome was captured using the Agilent SureSelect Human All Exon 50M exon capture kit and WES data was obtained by subsequent sequencing using the Illumina Hiseq2000 platform. The Burrows-Wheeler Aligner (BWA) [1] software was used to align the reads to the latest human genome reference sequence (hg19), filtering out reads which have low base quality bases (more than half of the bases which have a base quality of ≤ 5, including no calls) and/or with a mapping score of zero. Picard (http://picard.sourceforge.net) was used to mark duplicated reads and the alignment results were generated in BAM format. Single nucleotide polymorphisms (SNPs) were called using SOAPsnp [2] and small insertion/deletion events (indel) were detected by Samtools and GATK, and exported in VCF format [3-5]. VCF annotations were obtained from the Ensembl Variant Effect Predictor (VEP, for SNPs) [6] and Annovar (for indels) [7]. Predictions for missense mutations were obtained from FATHMM [8], SIFT [9], Polyphen-2 [10] and Condel [11]. Long runs of homozygosity larger than 5Mb (LRoH) were detected using Plink [12], and shorter ones (<5Mb) were detected manually using a custom Python script (input format: VCF) which plots homozygosity/heterozygosity state; and the resulting regions were converted to BED format (http://genome.ucsc.edu/FAQ/FAQformat.html#format1) to be viewed in IGV [13]. Initially we created two lists of genes to be reviewed as ‘prime candidates’ in the proband. The first one (hereafter called list 1, see PCD List 1) had all the known human PCD genes aforementioned.

The second (hereafter list 2, see PCD List 2) had all the genes (except known ones) in the Ciliome database (last updated: 24th Dec 2007 [14] plus a few additional genes which matched the keywords ‘dynein’, ‘radial spoke’, ‘nexin link’ and/or ‘cilia’ in the GeneCards website (www.genecards.org, v3.11) [15]. A separate analysis was done on all mutations with all genes included to ensure a non-biased analysis. Using the statistical software Stata (v13.1), VEP/Annovar annotations and VCF files were merged using the rsID as the key variable (a custom Python script was produced to assign an rsID which matches VEP output in the VCF files if missing). This enabled reviewing all ‘predicted high impact’ (PHI, hereafter Φ) mutations (i.e. very rare stop gains/losses, start losses, splice-site acceptor/donor variants, missense mutations, indels – both non-frameshifting and frameshifting) simultaneously which are homozygous and either are absent or very rare (<0.1%) in The Single Nucleotide Polymorphism Database (dbSNP) database [16]. The remaining mutations were then compared against our internal non-PCD patient database (previously whole-exome sequenced 13 individuals of Arabic ancestry), Exome Variant Server (EVS) [17] and 1000 Genomes Project (1000GP) [18] for their presence and minor allele frequency (MAF). The STRING software was used to predict the interactome of remaining candidate genes [19]. Additionally, a CNV analysis was carried out on the WES data using Control-FREEC [20].

Screening for c.924C>A in Saudi Arabian sample

DNA was extracted as abovementioned. PCR was used to amplify a region 221bp long (containing the stop gain loci) in these individuals (Supp. Table S1); and these fragments were digested using the AvrII enzyme (following manufacturer New England Biolabs’ protocol, catalogue no: R0174L) and viewed using 96-well microplate array diagonal gel electrophoresis (MADGE) [21] to check for the presence of the p.E309* mutation in the Saudi population. Nucleotide numbering system uses +1 as the A of the ATG translation initiation codon in the reference sequence, with the initiation codon (Met) as codon 1.

Electron Microscopy

Endoscopic nasal biopsy was taken from the posterior portion of the inferior turbinate. A piece of tissue measuring 2mm in (maximum) diameter was obtained and fixed in 2% buffered glutaraldehyde. Following fixation, the tissue was post-fixed in a buffered solution of osmium tetroxide in order to enhance the contrast.

For ultrastructural examination, the tissue was subsequently plastic embedded and ultrathin sections are cut using a diamond knife. The ultra-thin sections were mounted on a grid and sequentially stained by immersing the grid in solutions of lead citrate and urinyl acetate.
Semi-thin sections were cut at a thickness of 0.5-1 µm and stained with toluidine blue. The semi-thin sections were used to guide the selection of the area to be viewed in ultra-thin sections. The ultra-thin sections were then examined using the JEOL transmission Electron Microscope (model: JEM-1400).

Supp. Results

Whole-exome sequencing of proband

Total captured region was 118,507,605 base pairs (50,620,566 bases on target and 67,887,039 bases near target, the latter being flankings region within 200bp of exons). Coverage of target (i.e. exons) and flanking regions (e.g. introns, splice sites) was 98.2% and 92.7% respectively. The average sequencing depth on target was 61.49 and the fraction of target covered with at least 20 and 10 reads was 78.5% and 88.2% respectively (and >4 read depth = 94.4%). There were a total of 51,751,389 (high quality) reads with a mapping rate of 99.21%. All known PCD genes had similar figures and no idiosyncrasies were observed.

Motility of cilia

Although we cannot provide a video of cilia beating, our technicians have noted that >80% of the cilia were immotile in the tissue analysed.

Supp. References

1. Li, H. & Durbin, R. Fast and accurate short read alignment with Burrows–Wheeler transform. *Bioinformatics* **25**, 1754-1760 (2009).
2. Li, R. *et al.* SNP detection for massively parallel whole-genome resequencing. *Genome Res* **19**, 1124 - 1132 (2009).

3. McKenna, A. *et al.* The Genome Analysis Toolkit: a MapReduce framework for analyzing next-generation DNA sequencing data. *Genome Res* **20**, 1297 - 1303 (2010).

4. Li, H. The Sequence Alignment/Map format and SAMtools. *Bioinformatics* **25**, 2078-2079 (2009).

5. Danecek, P. *et al.* The variant call format and VCFtools. *Bioinformatics* **27**, 2156-8 (2011).

6. McLaren, W. *et al.* Deriving the consequences of genomic variants with the Ensembl API and SNP Effect Predictor. *Bioinformatics* **26**, 2069-2070 (2010).

7. Wang, K., Li, M. & Hakonarson, H. ANNOVAR: functional annotation of genetic variants from high-throughput sequencing data. *Nucleic Acids Res* **38**, e164 (2010).

8. Shihab, H.A. *et al.* Predicting the functional, molecular, and phenotypic consequences of amino acid substitutions using hidden markov models. *Hum Mutat* **34**: 57-65 (2013).

9. Ng, P. & Henikoff, S. SIFT: Predicting amino acid changes that affect protein function. *Nucleic Acids Res* **31**, 3812 - 3814 (2003).

10. Adzhubei, I.A. *et al.* A method and server for predicting damaging missense mutations. *Nat Meth* **7**, 248-249 (2010).

11. Gonzalez-Perez, A. & Lopez-Bigas, N. Improving the assessment of the outcome of nonsynonymous SNVs with a consensus deleteriousness score, Condel. *Am J Hum Genet* **88**, 440-9 (2011).

12. Purcell, S. *et al.* PLINK: a tool set for whole-genome association and population-based linkage analyses. *Am J Hum Genet* **81**, 559 - 575 (2007).

13. Thorvaldsdottir, H., Robinson, J.T. & Mesirov, J.P. Integrative Genomics Viewer (IGV): high-performance genomics data visualization and exploration. *Brief Bioinform* **14**, 178-92 (2013).

14. Inglis, P.N., Boroevich, K.A. & Leroux, M.R. Piecing together a ciliome. *Trends Genet* **22**, 491-500 (2006).

15. Safran, M. *et al.* GeneCards Version 3: the human gene integrator. *Database (Oxford)* **2010**, baq020 (2010).

16. Sherry, S.T. *et al.* dbSNP: the NCBI database of genetic variation. *Nucleic Acids Res* **29**, 308-11 (2001).
17. ESP, N.G. Exome Variant Server, NHLBI GO Exome Sequencing Project (ESP). Vol. 2013 (Web, 2013) http://evs.gs.washington.edu/EVS/.
18. Consortium, T.G.P. A map of human genome variation from population-scale sequencing. *Nature* **467**, 1061-1073 (2010).
19. Franceschini, A. *et al.* STRING v9.1: protein-protein interaction networks, with increased coverage and integration. *Nucleic Acids Res* **41**, D808-15 (2013).
20. Boeva, V. *et al.* Control-FREEC: a tool for assessing copy number and allelic content using next-generation sequencing data. *Bioinformatics* **28**, 423-5 (2012).
21. Day, I.N., Humphries, S.E., Richards, S., Norton, D. & Reid, M. High-throughput genotyping using horizontal polyacrylamide gels with wells arranged for microplate array diagonal gel electrophoresis (MADGE). *Biotechniques* **19**, 830-5 (1995).

PCD List 1

ENSG00000039139
ENSG00000122735
ENSG00000167646
ENSG00000154099
ENSG00000086288
ENSG00000105877
ENSG00000171595
ENSG00000165506
ENSG00000111834
ENSG00000172426
ENSG00000145075
ENSG00000141519
ENSG00000119661
ENSG00000167131
ENSG00000129295
ENSG00000157423
ENSG00000164818
ENSG00000046651
ENSG00000156313
ENSG00000105479
ENSG00000157856
ENSG00000139537
ENSG00000160188
ENSG00000256061
ENSG00000004838

PCD List 2

ENSG00000101052
ENSG00000114446
ENSG00000163879
ENSG00000068885
ENSG00000032742
ENSG00000138002
ENSG00000112530
ENSG00000145107
ENSG00000103351
ENSG00000163093
ENSG00000122970
ENSG00000130363
ENSG00000141013
ENSG00000105948
ENSG00000138587
ENSG00000109083
ENSG00000128581
ENSG00000167858
ENSG00000111837
ENSG00000184009
ENSG00000108641
ENSG00000196659
ENSG00000118096
ENSG00000146425
ENSG00000118997
ENSG00000128408
ENSG00000096093
ENSG00000162961
ENSG00000157796
ENSG00000123810
ENSG00000165533
ENSG00000168589
ENSG00000084207
ENSG00000102218
ENSG00000109971
ENSG00000125124
ENSG00000138175
ENSG00000075945
ENSG00000138036
ENSG00000150628
ENSG00000066185
ENSG00000152763
ENSG00000173093
ENSG00000182224
ENSG00000077327
ENSG00000173013
ENSG00000143156
ENSG00000158710
ENSG00000118965
ENSG00000070761
ENSG00000167552
ENSG00000113966
ENSG00000159713
ENSG00000187535
ENSG00000131233
ENSG00000185055
ENSG00000188229

ENSG00000123607
ENSG00000138686
ENSG00000173597
ENSG00000121083
ENSG00000158104
ENSG00000158079
ENSG00000154678
ENSG00000116885
ENSG00000109103
ENSG00000048342
ENSG00000164953
ENSG00000132005
ENSG00000150995
ENSG00000185246
ENSG00000163913
ENSG00000138160
ENSG00000090863
ENSG00000065183
ENSG00000160226
ENSG00000119927
ENSG00000152582
ENSG00000159079
ENSG00000141577
ENSG00000135205
ENSG00000151023
ENSG00000168625
ENSG00000166596
ENSG00000145491
ENSG00000164675
ENSG00000183833
ENSG00000163885
ENSG00000112981
ENSG00000161973
ENSG00000101222
ENSG00000103021
ENSG00000184154
ENSG00000163610
ENSG00000114841
ENSG00000080824
ENSG00000122507
ENSG00000173540
ENSG00000089101
ENSG00000158023
ENSG00000139428
ENSG00000007174
ENSG00000124074
ENSG00000096872
ENSG00000100360
ENSG00000146221
ENSG00000170385
ENSG00000176101
ENSG00000061918
ENSG00000099219
ENSG00000198730
ENSG00000165659
ENSG00000080608
ENSG00000129348
ENSG00000102125
ENSG00000152520
ENSG00000108094

ENSG00000163637
ENSG00000119929
ENSG00000188723
ENSG00000162441
ENSG00000151914
ENSG00000181610
ENSG00000106852
ENSG00000166165
ENSG00000011566
ENSG00000043514
ENSG00000152683
ENSG00000104907
ENSG00000054392
ENSG00000069974
ENSG00000013455
ENSG00000137349
ENSG00000130283
ENSG00000086758
ENSG00000196531
ENSG00000134265
ENSG00000198416
ENSG00000164252
ENSG00000163312
ENSG00000177889
ENSG00000180957
ENSG00000160967
ENSG00000091157
ENSG00000125875
ENSG00000126934
ENSG00000102221
ENSG00000187919
ENSG00000137200
ENSG00000104047
ENSG00000141342
ENSG00000122435
ENSG00000183690
ENSG00000100744
ENSG00000169299
ENSG00000085415
ENSG00000130348
ENSG00000077147
ENSG00000167972
ENSG00000010292
ENSG00000147576
ENSG00000168671
ENSG00000109133
ENSG00000198399
ENSG00000163001
ENSG00000134186
ENSG00000197930
ENSG00000148737
ENSG00000110756
ENSG00000142186
ENSG00000001036
ENSG00000146143
ENSG00000124193
ENSG00000166391
ENSG00000143995
ENSG00000135587
ENSG00000164885

ENSG00000188906
ENSG00000169359
ENSG00000125971
ENSG00000131873
ENSG00000147457
ENSG00000176749
ENSG00000012963
ENSG00000118873
ENSG00000143933
ENSG00000150457
ENSG00000117868
ENSG00000150316
ENSG00000179115
ENSG00000107937
ENSG00000131951
ENSG00000082068
ENSG00000171735
ENSG00000171316
ENSG00000075624
ENSG00000114480
ENSG00000198783
ENSG00000139116
ENSG00000124181
ENSG00000149187
ENSG00000164089
ENSG00000183597
ENSG00000101935
ENSG00000147224
ENSG00000118689
ENSG00000165097
ENSG00000117475
ENSG00000149792
ENSG00000132466
ENSG00000068394
ENSG00000168439
ENSG00000093217
ENSG00000122484
ENSG00000107036
ENSG00000079739
ENSG00000102898
ENSG00000167986
ENSG00000163655
ENSG00000198900
ENSG00000116030
ENSG00000168348
ENSG00000183048
ENSG00000145349
ENSG00000198626
ENSG00000182749
ENSG00000108176
ENSG00000163516
ENSG00000138081
ENSG00000136944
ENSG00000132437
ENSG00000197958
ENSG00000187778
ENSG00000197894
ENSG00000107223
ENSG00000123454
ENSG00000163378

ENSG00000100003
ENSG00000108592
ENSG00000115942
ENSG00000172409
ENSG00000089048
ENSG00000186298
ENSG00000038382
ENSG00000131242
ENSG00000159200
ENSG00000129347
ENSG00000164144
ENSG00000185009
ENSG00000064419
ENSG00000147604
ENSG00000105254
ENSG00000136485
ENSG00000090273
ENSG00000138678
ENSG00000165219
ENSG00000105568
ENSG00000196586
ENSG00000119698
ENSG00000110917
ENSG00000135636
ENSG00000090061
ENSG00000135472
ENSG00000162368
ENSG00000170889
ENSG00000125247
ENSG00000164587
ENSG00000175155
ENSG00000134313
ENSG00000189067
ENSG00000112514
ENSG00000105137
ENSG00000165152
ENSG00000146476
ENSG00000119865
ENSG00000171097
ENSG00000090861
ENSG00000172554
ENSG00000167658
ENSG00000156194
ENSG00000066136
ENSG00000154803
ENSG00000121068
ENSG00000155100
ENSG00000086827
ENSG00000151414
ENSG00000186676
ENSG00000100220
ENSG00000135018
ENSG00000008300
ENSG00000129159
ENSG00000127152
ENSG00000171435
ENSG00000182197
ENSG00000116833
ENSG00000117593
ENSG00000166402

ENSG00000060688
ENSG00000172046
ENSG00000085978
ENSG00000105519
ENSG00000108953
ENSG00000182768
ENSG00000008382
ENSG00000122507
ENSG00000160401
ENSG00000197826
ENSG00000010626
ENSG00000140057
ENSG00000197748
ENSG00000165990
ENSG00000104983
ENSG00000165695
ENSG00000132321
ENSG00000099889
ENSG00000135318
ENSG00000088727
ENSG00000156787
ENSG00000103599
ENSG00000167815
ENSG00000087302
ENSG00000116198
ENSG00000106479
ENSG00000164012
ENSG00000156042
ENSG00000112210
ENSG00000119333
ENSG00000101882
ENSG00000166855
ENSG00000109618
ENSG00000143493
ENSG00000089177
ENSG00000181378
ENSG00000168385
ENSG00000119650
ENSG00000119640
ENSG00000166024
ENSG00000089248
ENSG00000115459
ENSG00000149084
ENSG00000110711
ENSG00000100031
ENSG00000162643
ENSG00000115953
ENSG00000167977
ENSG00000183828
ENSG00000157106
ENSG00000152977
ENSG00000111727
ENSG00000158234
ENSG00000179632
ENSG00000113448
ENSG00000178662
ENSG00000165280
ENSG00000114473
ENSG00000179636
ENSG00000100246

ENSG00000164983
ENSG00000109323
ENSG00000135373
ENSG00000112541
ENSG00000081870
ENSG00000159720
ENSG00000146282
ENSG00000104321
ENSG00000100401
ENSG00000126226
ENSG00000120738
ENSG00000166183
ENSG00000142609
ENSG00000121350
ENSG00000115423
ENSG00000066382
ENSG00000132514
ENSG00000185760
ENSG00000141378
ENSG00000090054
ENSG00000149273
ENSG00000196118
ENSG00000035115
ENSG00000008952
ENSG00000173349
ENSG00000117395
ENSG00000119636
ENSG00000100129
ENSG00000079335
ENSG00000137522
ENSG00000112992
ENSG00000142168
ENSG00000167619
ENSG00000147400
ENSG00000169660
ENSG00000175110
ENSG00000108551
ENSG00000088247
ENSG00000095459
ENSG00000137161
ENSG00000145214
ENSG00000163714
ENSG00000150753
ENSG00000146872
ENSG00000171962
ENSG00000181085
ENSG00000122642
ENSG00000139318
ENSG00000173113
ENSG00000159556
ENSG00000168291
ENSG00000072786
ENSG00000137876
ENSG00000111880
ENSG00000048392
ENSG00000117713
ENSG00000154889
ENSG00000109339
ENSG00000115866
ENSG00000135414

ENSG00000095321
ENSG00000168028
ENSG00000145362
ENSG00000145782
ENSG00000121879
ENSG00000174444
ENSG00000075856
ENSG00000198718
ENSG00000128039
ENSG00000130508
ENSG00000131018
ENSG00000068784
ENSG00000128524
ENSG00000100567
ENSG00000136848
ENSG00000089195
ENSG00000117174
ENSG00000100413
ENSG00000148672
ENSG00000112759
ENSG00000151151
ENSG00000133703
ENSG00000104219
ENSG00000163945
ENSG00000082898
ENSG00000149577
ENSG00000141429
ENSG00000180185
ENSG00000104723
ENSG00000137992
ENSG00000137962
ENSG00000129187
ENSG00000127022
ENSG00000178802
ENSG00000163798
ENSG00000076003
ENSG00000117139
ENSG00000172009
ENSG00000164073
ENSG00000163788
ENSG00000198722
ENSG00000104888
ENSG00000148396
ENSG00000111364
ENSG00000071051
ENSG00000166311
ENSG00000182687
ENSG00000164934
ENSG00000186889
ENSG00000185008
ENSG00000134438
ENSG00000137413
ENSG00000095383
ENSG00000141570
ENSG00000151806
ENSG00000003509
ENSG00000113649
ENSG00000057468
ENSG00000187609
ENSG00000054116

ENSG00000111361
ENSG00000154035
ENSG00000103067
ENSG00000179292
ENSG00000135968
ENSG00000109536
ENSG00000155906
ENSG00000006717
ENSG00000113300
ENSG00000182858
ENSG00000154310
ENSG00000166206
ENSG00000164051
ENSG00000171863
ENSG00000175536
ENSG00000125877
ENSG00000160200
ENSG00000164329
ENSG00000132130
ENSG00000164815
ENSG00000091656
ENSG00000142798
ENSG00000182853
ENSG00000100997
ENSG00000073417
ENSG00000158062
ENSG00000099246
ENSG00000105220
ENSG00000101844
ENSG00000021776
ENSG00000071894
ENSG00000183291
ENSG00000178234
ENSG00000124383
ENSG00000102910
ENSG00000196262
ENSG00000080546
ENSG00000084623
ENSG00000116299
ENSG00000107593
ENSG00000135241
ENSG00000183780
ENSG00000130713
ENSG00000184408
ENSG00000183576
ENSG00000178952
ENSG00000165782
ENSG00000073584
ENSG00000124228
ENSG00000129625
ENSG00000144559
ENSG00000126698
ENSG00000198755
ENSG00000066651
ENSG00000140455
ENSG00000104331
ENSG00000166971
ENSG00000123395
ENSG00000053328
ENSG00000164109

ENSG00000177963
ENSG00000160799
ENSG00000165629
ENSG00000027001
ENSG00000168090
ENSG00000128607
ENSG00000188419
ENSG00000166224
ENSG00000137513
ENSG00000163161
ENSG00000158411
ENSG00000141543
ENSG00000145794
ENSG00000161513
ENSG00000146733
ENSG00000125037
ENSG00000184840
ENSG00000182087
ENSG00000166974
ENSG00000118046
ENSG00000106443
ENSG00000104412
ENSG00000074855
ENSG00000130313
ENSG00000096150
ENSG00000065665
ENSG00000184983
ENSG00000057608
ENSG00000133997
ENSG00000165406
ENSG00000146267
ENSG00000181029
ENSG00000140521
ENSG00000099964
ENSG00000088038
ENSG00000093000
ENSG00000156261
ENSG00000128923
ENSG00000131051
ENSG00000138663
ENSG00000156011
ENSG00000140443
ENSG00000138744
ENSG00000182544
ENSG00000173376
ENSG00000198910
ENSG00000165898
ENSG00000130165
ENSG00000148943
ENSG00000170836
ENSG00000121316
ENSG00000163697
ENSG00000104343
ENSG00000178252
ENSG00000010017
ENSG00000106263
ENSG00000140612
ENSG00000070423
ENSG00000197563
ENSG00000091164

ENSG00000167513
ENSG00000175193
ENSG00000198862
ENSG00000132424
ENSG00000105341
ENSG00000006451
ENSG00000115350
ENSG00000101096
ENSG00000103671
ENSG00000154059
ENSG00000103051
ENSG00000100601
ENSG00000159650
ENSG00000116690
ENSG00000156113
ENSG00000015133
ENSG00000136463
ENSG00000156873
ENSG00000197045
ENSG00000154380
ENSG00000163113
ENSG00000113141
ENSG00000165813
ENSG00000011426
ENSG00000114573
ENSG00000134255
ENSG00000089154
ENSG00000083307
ENSG00000157823
ENSG00000138641
ENSG00000121031
ENSG00000124207
ENSG00000106524
ENSG00000139684
ENSG00000121749
ENSG00000084092
ENSG00000068654
ENSG00000143748
ENSG00000196449
ENSG00000166925
ENSG00000114200
ENSG00000115468
ENSG00000088930
ENSG00000136891
ENSG00000166192
ENSG00000119718
ENSG00000157890
ENSG00000080189
ENSG00000114166
ENSG00000112474
ENSG00000135476
ENSG00000141385
ENSG00000137770
ENSG00000144591
ENSG00000072310
ENSG00000074047
ENSG00000065328
ENSG00000108055
ENSG00000108443
ENSG00000167475

ENSG00000198677
ENSG00000116649
ENSG00000175093
ENSG00000087299
ENSG00000162300
ENSG00000103642
ENSG00000086475
ENSG00000144959
ENSG00000131381
ENSG00000163428
ENSG00000197402
ENSG00000182827
ENSG00000113569
ENSG00000095794
ENSG00000113456
ENSG00000133019
ENSG00000118939
ENSG00000180917
ENSG00000127412
ENSG00000004487
ENSG00000162923
ENSG00000175575
ENSG00000171530
ENSG00000099904
ENSG00000165914
ENSG00000151148
ENSG00000165186
ENSG00000172613
ENSG00000157985
ENSG00000085840
ENSG00000077235
ENSG00000118971
ENSG00000120925
ENSG00000100226
ENSG00000110583
ENSG00000136319
ENSG00000134001
ENSG00000064313
ENSG00000134815
ENSG00000048544
ENSG00000143416
ENSG00000188878
ENSG00000171004
ENSG00000100296
ENSG00000090060
ENSG00000165671
ENSG00000130731
ENSG00000178105
ENSG00000132639
ENSG00000115541
ENSG00000197818
ENSG00000082701
ENSG00000088832
ENSG00000176476
ENSG00000153015
ENSG00000063854
ENSG00000089693
ENSG00000114331
ENSG00000105643
ENSG00000148200

ENSG00000164347
ENSG00000169925
ENSG00000160201
ENSG00000084072
ENSG00000168066
ENSG00000071189
ENSG00000129667
ENSG00000111142
ENSG00000126457
ENSG00000110063
ENSG00000106617
ENSG00000178913
ENSG00000120616
ENSG00000120008
ENSG00000171100
ENSG00000176890
ENSG00000132300
ENSG00000070061
ENSG00000110172
ENSG00000036257
ENSG00000090857
ENSG00000112379
ENSG00000136371
ENSG00000165704
ENSG00000198478
ENSG00000175287
ENSG00000180828
ENSG00000135821
ENSG00000169862
ENSG00000124641
ENSG00000160193
ENSG00000111530
ENSG00000113719
ENSG00000153107
ENSG00000116353
ENSG00000134049
ENSG00000115657
ENSG00000157426
ENSG00000198825
ENSG00000071082
ENSG00000122545
ENSG00000105401
ENSG00000138430
ENSG00000149485
ENSG00000002745
ENSG00000087095
ENSG00000058600
ENSG00000085999
ENSG00000104142
ENSG00000138083
ENSG00000174738
ENSG00000141127
ENSG00000185414
ENSG00000165304
ENSG00000109917
ENSG00000130396
ENSG00000075785
ENSG00000105993
ENSG00000165609
ENSG00000177076

ENSG00000181704
ENSG00000068323
ENSG00000173020
ENSG00000175220
ENSG00000005075
ENSG00000111186
ENSG00000055044
ENSG00000105655
ENSG00000172977
ENSG00000109101
ENSG00000100600
ENSG00000124772
ENSG00000165995
ENSG00000170854
ENSG00000186712
ENSG00000185721
ENSG00000076242
ENSG00000115561
ENSG00000033050
ENSG00000164172
ENSG00000111667
ENSG00000178878
ENSG00000178184
ENSG00000105135
ENSG00000130749
ENSG00000089094
ENSG00000115159
ENSG00000115825
ENSG00000156875
ENSG00000142657
ENSG00000100116
ENSG00000139352
ENSG00000100330
ENSG00000084733
ENSG00000144048
ENSG00000112685
ENSG00000104637
ENSG00000114126
ENSG00000103546
ENSG00000167325
ENSG00000196413
ENSG00000135404
ENSG00000105364
ENSG00000124541
ENSG00000059573
ENSG00000188818
ENSG00000175166
ENSG00000106348
ENSG00000183207
ENSG00000116151
ENSG00000084652
ENSG00000188677
ENSG00000166226
ENSG00000111674
ENSG00000141367
ENSG00000103335
ENSG00000135845
ENSG00000198408
ENSG00000149136
ENSG00000112592

ENSG00000018699
ENSG00000114346
ENSG00000187323
ENSG00000198931
ENSG00000145730
ENSG00000197121
ENSG00000010072
ENSG00000176009
ENSG00000187210
ENSG00000170004
ENSG00000176165
ENSG00000181789
ENSG00000158987
ENSG00000172238
ENSG00000049860
ENSG00000049656
ENSG00000137330
ENSG00000188869
ENSG00000157212
ENSG00000168906
ENSG00000169255
ENSG00000087470
ENSG00000177479
ENSG00000100380
ENSG00000168530
ENSG00000177093
ENSG00000120162
ENSG00000130055
ENSG00000170445
ENSG00000168818
ENSG00000165443
ENSG00000137474
ENSG00000111987
ENSG00000102189
ENSG00000110107
ENSG00000102743
ENSG00000106105
ENSG00000135624
ENSG00000166228
ENSG00000108091
ENSG00000115685
ENSG00000140859
ENSG00000162401
ENSG00000117054
ENSG00000003756
ENSG00000102312
ENSG00000135829
ENSG00000138279
ENSG00000104626
ENSG00000103356
ENSG00000182569
ENSG00000138326
ENSG00000133316
ENSG00000004961
ENSG00000167670
ENSG00000120705
ENSG00000127884
ENSG00000170312
ENSG00000105372
ENSG00000166598

ENSG00000083845
ENSG00000086061
ENSG00000120438
ENSG00000148225
ENSG00000095951
ENSG00000123201
ENSG00000072134
ENSG00000157540
ENSG00000146828
ENSG00000155438
ENSG00000128059
ENSG00000131375
ENSG00000197006
ENSG00000168724
ENSG00000143774
ENSG00000102144
ENSG00000166922
ENSG00000105245
ENSG00000079134
ENSG00000176658
ENSG00000151322
ENSG00000143476
ENSG00000080371
ENSG00000101890
ENSG00000152684
ENSG00000100219
ENSG00000086205
ENSG00000122378
ENSG00000085276
ENSG00000154945
ENSG00000137877
ENSG00000164764
ENSG00000079277
ENSG00000009694
ENSG00000117984
ENSG00000141646
ENSG00000143621
ENSG00000133313
ENSG00000163106
ENSG00000164062
ENSG00000169738
ENSG00000096717
ENSG00000100889
ENSG00000166341
ENSG00000153944
ENSG00000100297
ENSG00000153250
ENSG00000109189
ENSG00000100393
ENSG00000153481
ENSG00000185516
ENSG00000122954
ENSG00000184182
ENSG00000100823
ENSG00000164032
ENSG00000062650
ENSG00000156110
ENSG00000197872
ENSG00000099956
ENSG00000165672

ENSG00000131795
ENSG00000076650
ENSG00000108946
ENSG00000198721
ENSG00000163002
ENSG00000143499
ENSG00000163950
ENSG00000132970
ENSG00000159792
ENSG00000087460
ENSG00000105983
ENSG00000101210
ENSG00000184185
ENSG00000119950
ENSG00000182220
ENSG00000163623
ENSG00000185651
ENSG00000040933
ENSG00000110880
ENSG00000014138
ENSG00000171234
ENSG00000144061
ENSG00000100138
ENSG00000113851
ENSG00000138381
ENSG00000100983
ENSG00000104524
ENSG00000106038
ENSG00000075151
ENSG00000198060
ENSG00000077549
ENSG00000140043
ENSG00000136824
ENSG00000185973
ENSG00000071564
ENSG00000063438
ENSG00000157593
ENSG00000043591
ENSG00000163104
ENSG00000136758
ENSG00000152953
ENSG00000110046
ENSG00000133835
ENSG00000100523
ENSG00000070669
ENSG00000114423
ENSG00000156052
ENSG00000164506
ENSG00000113460
ENSG00000077097
ENSG00000196730
ENSG00000163357
ENSG00000100258
ENSG00000068796
ENSG00000166965
ENSG00000165417
ENSG00000115306
ENSG00000163873
ENSG00000134744
ENSG00000006042

ENSG00000138768
ENSG00000198001
ENSG00000077254
ENSG00000182400
ENSG00000108510
ENSG00000130383
ENSG00000138757
ENSG00000139131
ENSG00000196235
ENSG00000169764
ENSG00000142507
ENSG00000130702
ENSG00000181449
ENSG00000116957
ENSG00000165960
ENSG00000164609
ENSG00000198807
ENSG00000198746
ENSG00000183049
ENSG00000107669
ENSG00000074695
ENSG00000112339
ENSG00000172061
ENSG00000008256
ENSG00000196177
ENSG00000149091
ENSG00000087510
ENSG00000145826
ENSG00000171135
ENSG00000123094
ENSG00000009780
ENSG00000047578
ENSG00000140829
ENSG00000175137
ENSG00000198558
ENSG00000101152
ENSG00000101444
ENSG00000149571
ENSG00000099940
ENSG00000136783
ENSG00000155313
ENSG00000111405
ENSG00000132341
ENSG00000125352
ENSG00000152315
ENSG00000164933
ENSG00000115484
ENSG00000189308
ENSG00000166260
ENSG00000189007
ENSG00000120697
ENSG00000167740
ENSG00000083642
ENSG00000115216
ENSG00000101577
ENSG00000148688
ENSG00000114316
ENSG00000152234
ENSG00000133812
ENSG00000131781

ENSG00000099899
ENSG00000143256
ENSG00000103342
ENSG00000112208
ENSG00000112237
ENSG00000160007
ENSG00000153922
ENSG00000167004
ENSG00000069329
ENSG00000148154
ENSG00000131626
ENSG00000100023
ENSG00000183098
ENSG00000126016
ENSG00000162066
ENSG00000143314
ENSG00000108582
ENSG00000140400
ENSG00000178381
ENSG00000140463
ENSG00000132388
ENSG00000077782
ENSG00000198728
ENSG00000137343
ENSG00000160209
ENSG00000156976
ENSG00000125107
ENSG00000108439
ENSG00000146109
ENSG00000134899
ENSG00000174851
ENSG00000135776
ENSG00000129596
ENSG00000031544
ENSG00000185088
ENSG00000100592
ENSG00000127554
ENSG00000108829
ENSG00000132207
ENSG00000068793
ENSG00000138433
ENSG00000160220
ENSG00000112159
ENSG00000175745
ENSG00000167881
ENSG00000136643
ENSG00000123297
ENSG00000136478
ENSG00000072756
ENSG00000109670
ENSG00000040341
ENSG00000155878
ENSG00000183508
ENSG00000162298
ENSG00000114942
ENSG00000109654
ENSG00000182333
ENSG00000078403
ENSG00000154975
ENSG00000109163

ENSG00000105607
ENSG00000114982
ENSG00000074582
ENSG00000155304
ENSG00000168493
ENSG00000188244
ENSG00000147421
ENSG00000163468
ENSG00000183496
ENSG00000103769
ENSG00000104388
ENSG00000143158
ENSG00000101193
ENSG00000104325
ENSG00000179194
ENSG00000170734
ENSG00000147465
ENSG00000196132
ENSG00000079313
ENSG00000108296
ENSG00000174485
ENSG00000042088
ENSG00000033867
ENSG00000100417
ENSG00000133026
ENSG00000197885
ENSG00000140905
ENSG00000158825
ENSG00000141279
ENSG00000163636
ENSG00000084110
ENSG00000134909
ENSG00000184634
ENSG00000153933
ENSG00000104879
ENSG00000107815
ENSG00000106638
ENSG00000157916
ENSG00000125351
ENSG00000041982
ENSG00000139549
ENSG00000113558
ENSG00000129559
ENSG00000122687
ENSG00000131323
ENSG00000100266
ENSG00000120948
ENSG00000131238
ENSG00000129315
ENSG00000070081
ENSG00000159086
ENSG00000189338
ENSG00000166889
ENSG00000092108
ENSG00000172007
ENSG00000174276
ENSG00000100030
ENSG00000115241
ENSG00000169021
ENSG00000102003

ENSG00000145545
ENSG00000171152
ENSG00000077044
ENSG00000103319
ENSG00000161204
ENSG00000131236
ENSG00000182601
ENSG00000078140
ENSG00000121579
ENSG00000188167
ENSG00000089220
ENSG00000131013
ENSG00000159377
ENSG00000155111
ENSG00000151025
ENSG00000196656
ENSG00000112282
ENSG00000124208
ENSG00000173171
ENSG00000104695
ENSG00000133243
ENSG00000173366
ENSG00000177180
ENSG00000129255
ENSG00000159131
ENSG00000154027
ENSG00000109775
ENSG00000130255
ENSG00000001084
ENSG00000102038
ENSG00000089169
ENSG00000166377
ENSG00000144908
ENSG00000060069
ENSG00000170289
ENSG00000121644
ENSG00000197535
ENSG00000181222
ENSG00000115268
ENSG00000169718
ENSG00000011454
ENSG00000066583
ENSG00000180660
ENSG00000114742
ENSG00000103150
ENSG00000145780
ENSG00000138135
ENSG00000074621
ENSG00000118200
ENSG00000180875
ENSG00000169427
ENSG00000177084
ENSG00000188530
ENSG00000073803
ENSG00000088812
ENSG00000065978
ENSG00000108590
ENSG00000196911
ENSG00000179104
ENSG00000132436

ENSG00000165630
ENSG00000121957
ENSG00000006715
ENSG00000173011
ENSG00000152942
ENSG00000141485
ENSG00000188909
ENSG00000135473
ENSG00000175985
ENSG00000138193
ENSG00000148835
ENSG00000119616
ENSG00000165283
ENSG00000090686
ENSG00000055813
ENSG00000181982
ENSG00000188157
ENSG00000125820
ENSG00000109814
ENSG00000164327
ENSG00000167693
ENSG00000061676
ENSG00000176102
ENSG00000174227
ENSG00000101138
ENSG00000160271
ENSG00000155189
ENSG00000108424
ENSG00000072501
ENSG00000151240
ENSG00000197905
ENSG00000136854
ENSG00000140374
ENSG00000136868
ENSG00000171634
ENSG00000103544
ENSG00000131748
ENSG00000168876
ENSG00000113643
ENSG00000135940
ENSG00000133110
ENSG00000142910
ENSG00000104762
ENSG00000159461
ENSG00000148690
ENSG00000103241
ENSG00000114503
ENSG00000196636
ENSG00000163638
ENSG00000145414
ENSG00000145331
ENSG00000183617
ENSG00000067225
ENSG00000115641
ENSG00000143106
ENSG00000143303
ENSG00000089157
ENSG00000140319
ENSG00000125755
ENSG00000144406

ENSG00000101391
ENSG00000197329
ENSG00000145020
ENSG00000152689
ENSG00000115504
ENSG00000120318
ENSG00000163541
ENSG00000013275
ENSG00000125954
ENSG00000150768
ENSG00000177602
ENSG00000129521
ENSG00000122008
ENSG00000103423
ENSG00000110025
ENSG00000117222
ENSG00000073910
ENSG00000172663
ENSG00000039650
ENSG00000013375
ENSG00000112489
ENSG00000183760
ENSG00000139697
ENSG00000140995
ENSG00000057149
ENSG00000114204
ENSG00000160294
ENSG00000036054
ENSG00000160325
ENSG00000101350
ENSG00000147416
ENSG00000197890
ENSG00000155052
ENSG00000175467
ENSG00000104299
ENSG00000070010
ENSG00000090621
ENSG00000175344
ENSG00000137335
ENSG00000120800
ENSG00000138246
ENSG00000115234
ENSG00000162104
ENSG00000136100
ENSG00000147509
ENSG00000141556
ENSG00000140015
ENSG00000095564
ENSG00000104375
ENSG00000125505
ENSG00000007372
ENSG00000148175
ENSG00000115275
ENSG00000177058
ENSG00000166321
ENSG00000133731
ENSG00000132694
ENSG00000198026
ENSG00000100316
ENSG00000004139

ENSG00000106628
ENSG00000176986
ENSG00000143105
ENSG00000131504
ENSG00000177628
ENSG00000162733
ENSG00000115211
ENSG00000160917
ENSG00000126062
ENSG00000004866
ENSG00000103274
ENSG00000178921
ENSG00000172915
ENSG00000133773
ENSG00000163848
ENSG00000182899
ENSG00000109736
ENSG00000101425
ENSG00000094880
ENSG00000149483
ENSG00000147403
ENSG00000135972
ENSG00000105223
ENSG00000115649
ENSG00000013503
ENSG00000163703
ENSG00000147471
ENSG00000119523
ENSG00000140575
ENSG00000105438
ENSG00000099204
ENSG00000174165
ENSG00000196642
ENSG00000108587
ENSG00000140519
ENSG00000164100
ENSG00000102786
ENSG00000107147
ENSG00000007062
ENSG00000184545
ENSG00000100262
ENSG00000131732
ENSG00000114541
ENSG00000111581
ENSG00000075415
ENSG00000146731
ENSG00000029725
ENSG00000109472
ENSG00000198183
ENSG00000196975
ENSG00000184701
ENSG00000178934
ENSG00000178104
ENSG00000178053
ENSG00000177664
ENSG00000167553
ENSG00000164111
ENSG00000163191
ENSG00000152076
ENSG00000137285

ENSG00000133665
ENSG00000127952
ENSG00000124688
ENSG00000117477
ENSG00000117266
ENSG00000114353
ENSG00000112964
ENSG00000106012
ENSG00000104833
ENSG00000104237
ENSG00000096238
ENSG00000092820
ENSG00000075142
ENSG00000064199
ENSG00000014216
ENSG00000184874
ENSG00000089472
ENSG00000169126
ENSG00000054267
ENSG00000104450
ENSG00000163808
ENSG00000139323
ENSG00000156876
ENSG00000114395
ENSG00000109576
ENSG00000104872
ENSG00000078687
ENSG00000108825
ENSG00000084693
ENSG00000110321
ENSG00000155761
ENSG00000131981
ENSG00000133739
ENSG00000104313
ENSG00000198782
ENSG00000181045
ENSG00000153904
ENSG00000055955
ENSG00000162814
ENSG00000138892
ENSG00000108046
ENSG00000110429
ENSG00000183773
ENSG00000101350
ENSG00000101350
ENSG00000092850
ENSG00000182247
ENSG00000165886
ENSG00000164414
ENSG00000105982
ENSG00000123360
ENSG00000197816
ENSG00000185681
ENSG00000162971
ENSG00000177042
ENSG00000188659
ENSG00000197746
ENSG00000105792
ENSG00000060138
ENSG00000088986

ENSG00000167578
ENSG00000165868
ENSG00000120262
ENSG00000178125
ENSG00000163060
ENSG00000163576
ENSG00000155530
ENSG00000100565
ENSG00000157227
ENSG00000135338
ENSG00000164323
ENSG00000139496
ENSG00000079974
ENSG00000135702
ENSG00000116863
ENSG00000120647
ENSG00000161996
ENSG00000138032
ENSG00000196562
ENSG00000132321
ENSG00000135108
ENSG00000163728
ENSG00000100218
ENSG00000109680
ENSG00000139714
ENSG00000102531
ENSG00000138669
ENSG00000134376
ENSG00000139154
ENSG00000198089
ENSG00000164627
ENSG00000198003
ENSG00000132275
ENSG00000048342
ENSG00000151704
ENSG00000117450
ENSG00000166445
ENSG00000100583
ENSG00000100271
ENSG00000132677
ENSG00000006468
ENSG00000115524
ENSG00000175175
ENSG00000115947
ENSG00000104320
ENSG00000166323
ENSG00000136878
ENSG00000172070
ENSG00000115548
ENSG00000133114
ENSG00000118307
ENSG00000165698
ENSG00000164651
ENSG00000180715
ENSG00000136319
ENSG00000169402
ENSG00000135778
ENSG00000167210
ENSG00000115137
ENSG00000164070

ENSG00000008086
ENSG00000109846
ENSG00000164619
ENSG00000176273
ENSG00000151849
ENSG00000170502
ENSG00000114859
ENSG00000105640
ENSG00000085231
ENSG00000110060
ENSG00000135334
ENSG00000134146
ENSG00000023909
ENSG00000137845
ENSG00000187109
ENSG00000115652
ENSG00000165195
ENSG00000152348
ENSG00000084774
ENSG00000198756
ENSG00000055130
ENSG00000099795
ENSG00000170522
ENSG00000063761
ENSG00000173599
ENSG00000175390
ENSG00000155961
ENSG00000112110
ENSG00000096401
ENSG00000112658
ENSG00000122591
ENSG00000165416
ENSG00000134748
ENSG00000163491
ENSG00000039319
ENSG00000198833
ENSG00000125814
ENSG00000130720
ENSG00000124614
ENSG00000145088
ENSG00000131473
ENSG00000101890
ENSG00000172680
ENSG00000160505
ENSG00000160629
ENSG00000118482
ENSG00000099769
ENSG00000072110
ENSG00000164344
ENSG00000132139
ENSG00000137808
ENSG00000105771
ENSG00000125409
ENSG00000103160
ENSG00000135387
ENSG00000181322
ENSG00000139620
ENSG00000158486
ENSG00000112081
ENSG00000198498

ENSG00000100982
ENSG00000176410
ENSG00000175104
ENSG00000171160
ENSG00000122692
ENSG00000137691
ENSG00000148356
ENSG00000177225
ENSG00000133710
ENSG00000104044
ENSG00000184260
ENSG00000110002
ENSG00000101624
ENSG00000011143
ENSG00000133110
ENSG00000198677
ENSG00000012048
ENSG00000080572
ENSG00000100285
ENSG00000155636
ENSG00000070961
ENSG00000001630
ENSG00000119125
ENSG00000186063
ENSG00000159307
ENSG00000151014
ENSG00000185518
ENSG00000118017
ENSG00000066279
ENSG00000166169
ENSG00000130706
ENSG00000121057
ENSG00000134717
ENSG00000113597
ENSG00000101421
ENSG00000155970
ENSG00000089050
ENSG00000124275
ENSG00000130772
ENSG00000138231
ENSG00000102580
ENSG00000175066
ENSG00000006712
ENSG00000118518
ENSG00000164074
ENSG00000135862
ENSG00000162390
ENSG00000065150
ENSG00000187773
ENSG00000112699
ENSG00000165650
ENSG00000110628
ENSG00000198162
ENSG00000161057
ENSG00000125743
ENSG00000054523
ENSG00000158006
ENSG00000120907
ENSG00000170820
ENSG00000176715

ENSG00000176201
ENSG00000185130
ENSG00000181830
ENSG00000108848
ENSG00000157445
ENSG00000089685
ENSG00000143079
ENSG00000140988
ENSG00000179580
ENSG00000120868
ENSG00000173546
ENSG00000186417
ENSG00000132911
ENSG00000182831
ENSG00000177971
ENSG00000164187
ENSG00000130985
ENSG00000180817
ENSG00000134109
ENSG00000031698
ENSG00000175595
ENSG00000185418
ENSG00000141627
ENSG00000196367
ENSG00000126005
ENSG00000085563
ENSG00000104687
ENSG00000060237
ENSG00000149292
ENSG00000100300
ENSG00000105865
ENSG00000144182
ENSG00000066279
ENSG00000169174
ENSG00000100324
ENSG00000127481
ENSG00000107130
ENSG00000004766
ENSG00000006625
ENSG00000110801
ENSG00000176261
ENSG00000171307
ENSG00000182473
ENSG00000172732
ENSG00000111110
ENSG00000123427
ENSG00000172062
ENSG00000167136
ENSG00000147677
ENSG00000112701
ENSG00000178607
ENSG00000106689
ENSG00000116198
ENSG00000136449
ENSG00000135315
ENSG00000174903
ENSG00000135720
ENSG00000188620
ENSG00000113722
ENSG00000165322

ENSG00000106268
ENSG00000113013
ENSG00000173145
ENSG00000100439
ENSG00000100664
ENSG00000180979
ENSG00000085721
ENSG00000168129
ENSG00000136875
ENSG00000102978
ENSG00000005206
ENSG00000170941
ENSG00000167632
ENSG00000160293
ENSG00000077721
ENSG00000163517
ENSG00000130227
ENSG00000100207
ENSG00000151079
ENSG00000153936
ENSG00000161202
ENSG00000116337
ENSG00000113282
ENSG00000162992
ENSG00000164114
ENSG00000016391
ENSG00000196365
ENSG00000113312
ENSG00000173811
ENSG00000185141
ENSG00000163624
ENSG00000197114
ENSG00000198929
ENSG00000165105
ENSG00000182786
ENSG00000128609
ENSG00000167701
ENSG00000003147
ENSG00000163877
ENSG00000073921
ENSG00000176887
ENSG00000155158
ENSG00000110066
ENSG00000138696
ENSG00000166275
ENSG00000058600
ENSG00000151576
ENSG00000175344
ENSG00000023318
ENSG00000067829
ENSG00000173826
ENSG00000091010
ENSG00000172469
ENSG00000105835
ENSG00000134321
ENSG00000076108
ENSG00000151575
ENSG00000133393
ENSG00000166333
ENSG00000004455

ENSG00000164898
ENSG00000105486
ENSG00000078177
ENSG00000137628
ENSG00000151790
ENSG00000138190
ENSG00000175792
ENSG00000075568
ENSG00000142700
ENSG00000165282
ENSG00000135638
ENSG00000114686
ENSG00000174013
ENSG00000163939
ENSG00000143486
ENSG00000167770
ENSG00000123728
ENSG00000092020
ENSG00000107862
ENSG00000081721
ENSG00000067167
ENSG00000148459
ENSG00000115252
ENSG00000165905
ENSG00000138303
ENSG00000132541
ENSG00000115593
ENSG00000178695
ENSG00000126432
ENSG00000116096
ENSG00000160993
ENSG00000135049
ENSG00000117151
ENSG00000103042
ENSG00000104808
ENSG00000137171
ENSG00000120440
ENSG00000151715
ENSG00000082153
ENSG00000174996
ENSG00000152932
ENSG00000116001
ENSG00000196433
ENSG00000129071
ENSG00000143199
ENSG00000187908
ENSG00000116685
ENSG00000131100
ENSG00000162392
ENSG00000159409
ENSG00000160439
ENSG00000100364
ENSG00000116035
ENSG00000163785
ENSG00000110700
ENSG00000086102
ENSG00000148843
ENSG00000115919
ENSG00000100902
ENSG00000070785

ENSG00000163956
ENSG00000174576
ENSG00000101346
ENSG00000179761
ENSG00000163818
ENSG00000095596
ENSG00000101057
ENSG00000164118
ENSG00000170927
ENSG00000179889
ENSG00000177885
ENSG00000118194
ENSG00000044446
ENSG00000116663
ENSG00000171824
ENSG00000132676
ENSG00000129932
ENSG00000179598
ENSG00000168079
ENSG00000112498
ENSG00000008869
ENSG00000107902
ENSG00000101557
ENSG00000156869
ENSG00000080503
ENSG00000161328
ENSG00000178202
ENSG00000153575
ENSG00000135677
ENSG00000167118
ENSG00000142208
ENSG00000188329
ENSG00000127980
ENSG00000064999
ENSG00000165118
ENSG00000168016
ENSG00000185798
ENSG00000122705
ENSG00000126778
ENSG00000120265
ENSG00000170426
ENSG00000174327
ENSG00000141564
ENSG00000168738
ENSG00000145782
ENSG00000151849
ENSG00000116747
ENSG00000156172
ENSG00000188010
ENSG00000137343
ENSG00000165502
ENSG00000103494
ENSG00000132768
ENSG00000074319
ENSG00000175938
ENSG00000105426
ENSG00000119686
ENSG00000106211
ENSG00000068383
ENSG00000159063

ENSG00000115677
ENSG00000112333
ENSG00000156502
ENSG00000142230
ENSG00000088035
ENSG00000197976
ENSG00000163539
ENSG00000112498
ENSG00000059758
ENSG00000182010
ENSG00000089060
ENSG00000100284
ENSG00000185624
ENSG00000155666
ENSG00000149260
ENSG00000163808
ENSG00000100575
ENSG00000167393
ENSG00000023608
ENSG00000177000
ENSG00000136842
ENSG00000164860
ENSG00000028528
ENSG00000121989
ENSG00000177666
ENSG00000115514
ENSG00000107959
ENSG00000160179
ENSG00000183826
ENSG00000164244
ENSG00000083520
ENSG00000196151
ENSG00000161326
ENSG00000139239
ENSG00000069399
ENSG00000135392
ENSG00000105011
ENSG00000113712
ENSG00000141551
ENSG00000110514
ENSG00000153558
ENSG00000150760
ENSG00000146242
ENSG00000106976
ENSG00000166164
ENSG00000124275
ENSG00000198690
ENSG00000171055
ENSG00000128694
ENSG00000109062
ENSG00000005810
ENSG00000155957
ENSG00000100033
ENSG00000007392
ENSG00000149823
ENSG00000144579
ENSG00000152404
ENSG00000151247
ENSG00000091009
ENSG00000143761

ENSG00000130150
ENSG00000125691
ENSG00000131373
ENSG00000139719
ENSG00000109686
ENSG00000168591
ENSG00000111596
ENSG00000128050
ENSG00000138604
ENSG00000167258
ENSG00000153574
ENSG00000137955
ENSG00000107731
ENSG00000143815
ENSG00000140632
ENSG00000054356
ENSG00000163354
ENSG00000144136
ENSG00000120586
ENSG00000112062
ENSG00000101266
ENSG00000160211
ENSG00000100416
ENSG00000010244
ENSG00000171603
ENSG00000091127
ENSG00000169696
